# Comprehensive strategies and measures to control COVID-19

**DOI:** 10.1186/s40249-022-00994-w

**Published:** 2022-06-11

**Authors:** Shun-Xian Zhang, Ming Yang, Jin-Xin Zheng, Bin-Qian Zhang, Chen-Hui Pan, Li-Guang Tian

**Affiliations:** 1 Longhua Hospital, Shanghai University of Traditional Chinese Medicine, Shanghai, 200032 China; 2 School of Global Health, Chinese Center for Tropical Diseases Research-Shanghai Jiao Tong University School of Medicine, Shanghai, 200025 China

## Background

Infectious diseases pandemic can lead to explosive effect with unpredictability on the world, as exemplified the bubonic–pneumonic plague pandemic in the fourteenth century [1], the 1918 influenza pandemic and the coronavirus disease 2019 (COVID-19) pandemic caused by severe acute respiratory syndrome coronavirus 2 (SARS-CoV-2). From January 2020 to May 2022, a total of 527.5 million individuals were suffered from an COVID-19, and more than 6.2 million individuals were died [2]. The global all-age rate of excess mortality due to the COVID-19 pandemic was 120.3 deaths per 100,000 [3]. Its threat to human beings, especially those with underlying health issues, no one overlook this outcome.

## The principles for forming comprehensive strategies and measures

The successes in response to COVID-19 threats have come not just from scientific cognition of disease characteristics (SARS-CoV-2) strain biological characteristics, disease prognosis, etc., but also from broad approaches that play a complementary role to fight against COVID-19, including constant surveillance of SARS-CoV-2 strain, clinical and public health efforts, and efficient translation of new findings into disease-control application and implementation.

The comprehensive strategies and measures are formulated based on four elements [4]: (1) SARS-CoV-2 strain characteristics include infectivity, pathogenicity, and mutations. (2) Social and economic development situation, including demographic characteristics, medical resource, material supply, etc. (3) Culture, scientific and technological level. (4) Government will, prevention and control concept, social system and social mobilization capacity.

## Beneficial experiences in practice and theory help to form effective epidemic-control strategies and measures

**Fig.1:**
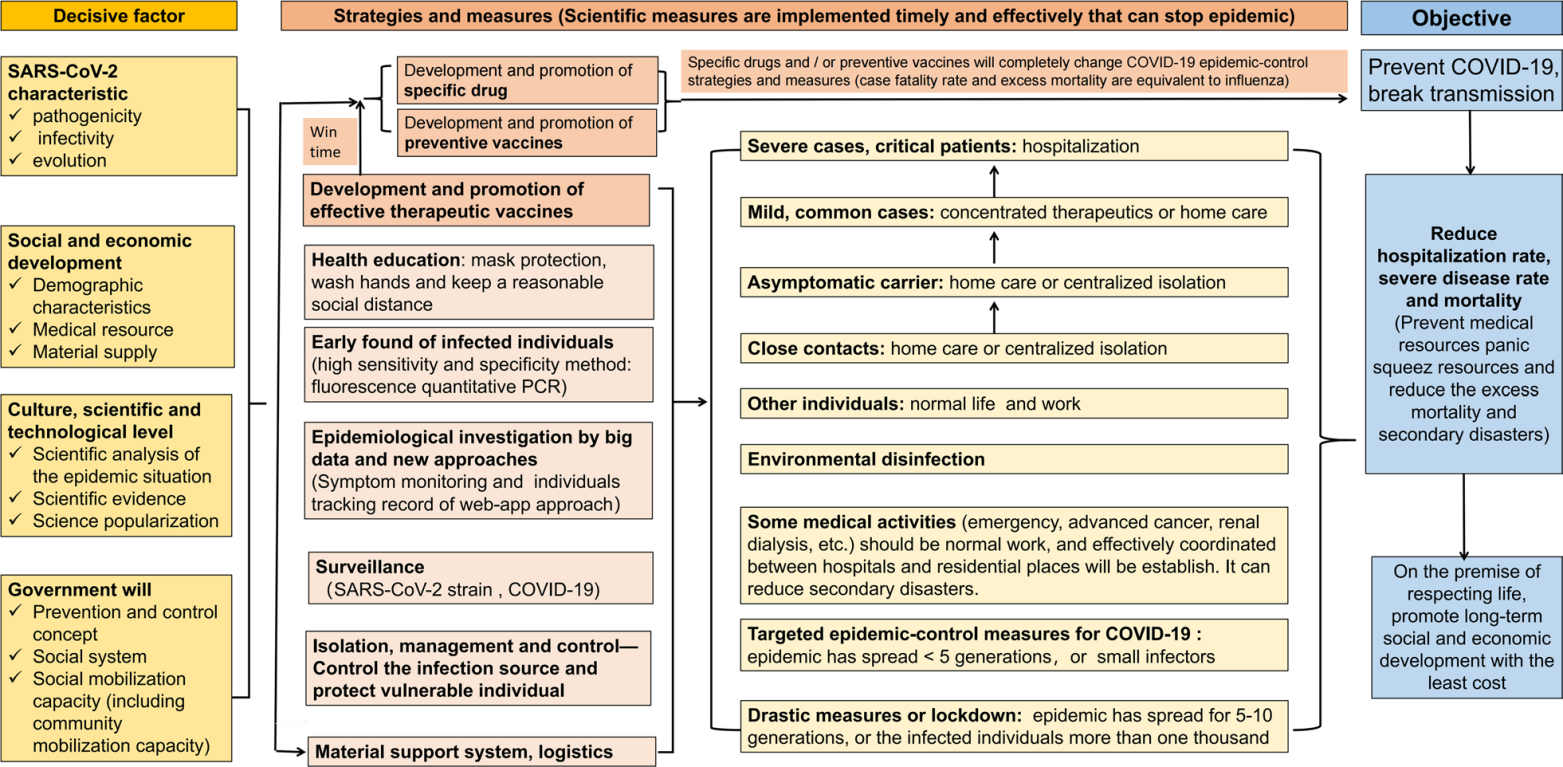
The epidemic-control strategies and measures for COVID-19

Formulating epidemic-control strategies will be complied with some beneficial experiences (Fig. 1), such as (1) Pathogen mutation surveillance. (2) Nucleic acid testing and antigen detection will be used to find infectors, coordinate with precise epidemiological investigation with big data, it can find infectors and stop community transmission. (3) Asymptomatic infectious individuals accelerate COVID-19 community spread and contribute magnitude cases. (4) Infectors will be immediately transferred to cabin hospital or designated hospitals for health care, and close contact tracing could both play role in blocking the transmission and identifying infectors, in addition, infector can be managed by classification, it can not only decrease severe cases, but also reduce the outflow of medical resources. (5) Breakthrough infection after vaccination prove that the vaccine cannot produce lasting protection. Developing effective preventive vaccine or broad-spectrum vaccine is still one of the most important tasks. (6) Stage-based processes and expansion phases of SARS-CoV-2 transmission is similar to biological invasion [5]. Hence, cross-disciplinary invasion science offers valuable insights that can facilitate control policy.

## The targeted intervention program can stop the COVID-19 spread

In response to suppress transmission, the first priority is to quickly find infectors, it is achieved by nucleic acid testing, application of big data in epidemiological investigation and the cooperation and public support. Epidemic transmission in small-scale can be controlled with targeted epidemic-control measures. However when the epidemic has spread and abundant infected individuals have been produced, strong measures must be taken to stop the rapid communication [5]. In fact, implementation of scientific measures can help us coordinate epidemic control and economic and social development, it can effectively block COVID-19 disseminate in small scope or in the early stage of the epidemic, it can minimize the impact of the epidemic and the number of infections, severe cases and deaths. Ultimately, it can control epidemic in the shortest time.

## Conclusions

COVID-19 transmission are driven by ecological and socioeconomic factors, scientific evidence from theory and interventions guide future policy making, the targeted epidemic-control COVID-19 need sufficient medical resources, strong social mobilization and civic cooperation.

## Data Availability

Not applicable.

## Abbreviations

COVID-19: Coronavirus disease 2019
SARS-COV-2: Severe acute respiratory syndrome coronavirus 2
